# Vascular disease in COPD: Systemic and pulmonary expression of PARC (Pulmonary and Activation-Regulated Chemokine)

**DOI:** 10.1371/journal.pone.0177218

**Published:** 2017-05-18

**Authors:** Mariana Muñoz-Esquerre, Elisabet Aliagas, Marta López-Sánchez, Ignacio Escobar, Daniel Huertas, Rosa Penín, Jordi Dorca, Salud Santos

**Affiliations:** 1 Department of Pulmonary Medicine, Bellvitge University Hospital -IDIBELL, University of Barcelona, L’Hospitalet de Llobregat, Barcelona, Spain; 2 Department of Thoracic Surgery, Bellvitge University Hospital -IDIBELL, University of Barcelona, L’Hospitalet de Llobregat, Barcelona, Spain; 3 Department of Pathology, Bellvitge University Hospital -IDIBELL, University of Barcelona, L’Hospitalet de Llobregat, Barcelona, Spain; 4 Biomedical Research Networking Center Consortium -Respiratory Diseases (CIBERES), Barcelona, Spain; Forschungszentrum Borstel Leibniz-Zentrum fur Medizin und Biowissenschaften, GERMANY

## Abstract

**Introduction:**

The role of Pulmonary and Activation-Regulated Chemokine (PARC) in the physiopathology of Chronic Obstructive Pulmonary Disease (COPD) is not fully understood. The aim of the present study is to analyze the expression of PARC in lung tissue and its relationship with the vascular remodeling of the systemic and pulmonary arteries of COPD subjects.

**Methods:**

To achieve this objective, protein and gene expression experiments, together with ELISA assays, were performed on the lung tissue, intercostal arteries and serum samples from COPD patients, non-obstructed smokers (NOS) and never-smokers (NS).

**Results:**

A total of 57 subjects were included in the analysis (23 COPD, 18 NOS and 16 NS). In the comparisons between groups, a significantly increased lung protein expression of PARC was observed in the COPD group compared to the NOS group (1.96±0.22 vs. 1.29±0.27, P-adjusted = 0.038). PARC was located predominantly in the smooth muscle cells of the remodeled pulmonary muscular arteries and the macrophage-rich area of the alveolar parenchyma. No differences were detected in PARC gene expression analyses. The protein content of PARC in the intercostal arteries were similar between groups, though little remodeling was observed in these arteries. Circulating levels of PARC were numerically higher in patients with COPD compared to NOS and NS.

**Conclusion:**

The results of the present study suggest an increased lung protein expression of PARC in COPD subjects. This protein was mainly localized in the smooth muscle cells of the pulmonary muscular arteries and was associated with the severity of intimal thickening, indicating its possible role in this remodeling process.

## Introduction

Chronic Obstructive Pulmonary Disease (COPD) is characterized by an abnormal inflammatory response of the lungs to noxious particles or gases, particularly cigarette smoke [1–2].Cardiovascular disease (CVD) is the most important comorbidity associated with COPD, due to its impact on patients’ overall prognosis, including mortality [1,3–5].It has been suggested that this systemic inflammation may be one of the main factors that play a significant role in the pathogenesis of atherothrombosis in COPD [6–7]. In this setting, chemokines are a group of chemotactic molecules that appear to regulate the directed movement of leukocytes and may therefore play important roles in inflammation and immunity [8].Of particular interest is the Pulmonary and Activation-Regulated Chemokine (PARC), also known as CCL18 or Macrophage Inflammatory Protein-4 (MIP-4), which is a new member of the CC chemokine family [8]. Although early studies described the constitutive lung tissue expression of PARC in humans [8–9], the role of PARC in the physiopathology of COPD and its relationship with the systemic vascular involvement described in this chronic condition are currently unknown [10].A small number of studies suggest that PARC could be a serum biomarker of cardiovascular mortality in large populations of COPD patients [11–12]. However, to the best of our knowledge, there are no previous data directly addressing the tissue characterization of PARC in COPD. Therefore, the current hypothesis was that PARC expression could be modified in COPD. The aim of the present study was to analyze the expression of PARC at the pulmonary, systemic and circulatory levels in the context of this respiratory disease. To achieve this objective, protein and gene expression experiments, together with ELISA assays, were performed on lung, intercostal (IC) artery and serum samples from COPD patients, non-obstructed smokers (NOS) and never-smokers (NS).The correlation between the immunostaining of PARC in both tissues (lung and systemic arteries) and their intimal thickening were studied.

## Materials and methods

### Subjects

This was a prospective study, performed in consecutive subjects who underwent lung resection (lobectomy or pneumonectomy) for the treatment of localized primary lung cancer. In line with the current definition of COPD in the GOLD guidelines [1],patients were divided into three groups: 1) COPD subjects (all of them current or former smokers), 2) non-obstructed smokers (NOS), and 3) never-smokers (NS). All procedures were perform in accordance with the Declaration of Helsinki, and protocols were approved by the local ethics committee “Comitè Ètic d’ Investigació Clínica del Hospital de Bellvitge, N° PR006/11”, An informed consent form was obtained from all participants.

### Sample collection

Lung specimens, sections of the 5th posterior IC artery and venous blood samples, were collected from all subjects. All lung tissue samples were obtained at a minimum distance of 5 cm from the tumor localization. Tissues samples were fixed overnight in 4% paraformaldehyde and embedded in paraffin. A microscopic evaluation was performed on the lung tissue to confirm the absence of neoplastic cells before it was included in the analysis. Venous blood samples were collected prior to surgery. Serum was extracted and stored at -80°C until it was used.

### Western blot assays

The PARC protein expression of lung and IC artery was examined by western blot analysis. Protein concentrations were measured using the Lowry method. In short, 40μg of lung and 50μg of IC artery protein homogenates were loaded to pre-cast 4–20% polyacrylamide-SDS gradient gel for electrophoresis and then transferred onto nitrocellulose membranes (Bio-Rad, Hercules, CA, USA). The membranes were blocked in tris buffer saline containing 0.1% Tween® 20 (TBS-T) and 5% bovine serum albumin (pH 7.4) for 1.5 hours (h) at room temperature (RT). The primary antibody against PARC was incubated at RT for 1hour (1/1000, AB104867, Abcam, Cambridge, UK). After 3 washes with TBS-T, membranes were incubated for 1h with polyclonal goat anti-rabbit horseradish peroxidase-conjugated secondary antibody (1/2000; Dako, Carpinteria, CA, USA). The Clarity Western ECL System (Bio-Rad) was used to detect the protein signal. Results were digitized using the Image Reader LAS-3000 (Fujifilm, Tokyo, Japan). Band density was quantified by densitometry using Multi-gauge v1.3 software and normalized to β-actin levels (AB8226, Abcam).

### Immunolabeling experiments

Immunohistochemistry and immunofluorescence experiments were carried out in order to study the expression and the exact localization of PARC in both tissues (lung and systemic arteries). Briefly, paraformaldehyde fixed paraffin embedded tissue sections of 4 μm were deparaffinised, rehydrated and rinsed in phosphate buffer saline (PBS). Antigen retrieval was for 1 minute at 100°C using a citrate buffer, pH 6. After 3 rinses in PBS, tissue sections were pre-incubated for 2 hours at RT in 20% normal goat serum (Gibco, Paisley, UK), 0.2% gelatin (Merck, Darmstadt, Germany) and 0.1% triton^®^ X-100 (Sigma-Aldrich, Sant Louis, Missouri, MO, USA). Slices were then incubated overnight at 4°C with the following primary antibodies: rabbit anti-human MIP-4 (1/400, Peprotech, Rocky Hill, New Jersey, NJ, USA), and in fluorescence experiments, rabbit anti-human MIP-4 (1/250, Abcam) or mouse anti-human alpha smooth muscle actin (αSMA) clone 1A4 as a smooth muscle cells (SMC) marker (1/400, A 5228, Sigma). After three washes in PBS-triton, samples were incubated with the avidin-biotin complex/peroxidase (Vectastain Elite ABC kit, Vector Laboratories, Burlingame, CA, USA) or, in fluorescent assays, with Alexa Fluor 488- or 555-goat anti-mouse or anti-rabbit (Life technologies, Paisley, UK) for 1 h at RT. Nuclei were counterstained with haematoxylin or, alternatively, in fluorescent assays, To-Pro^®^-3 (Life technologies) were used to visualize the nuclei. The results were observed and photographed under Leica DMD 108 light microscope (Leica Microsystems, Wetzlar, Germany) or, in fluorescence assays, under a Leica TCS-SL spectral confocal microscope (Leica).

Immunohistochemistry evaluation was performed in all samples and was used to semi-quantify the PARC expression in both tissues. Two blind observers performed the analysis of the following structures: 1) muscular pulmonary arteries (external diameter between 100 and 500 micrometers), 2) alveolar parenchyma, 3) bronchus and 4) IC arteries. In both pulmonary muscular and IC arteries, the labeling of the intima, medial and adventitia layers was assessed. The bronchial evaluation included epithelial cell layer, sub-epithelial baseline membrane (SEBM), and airway SMCs. Label intensity was scored as negative (0), mild (1), moderate (2), or strongly positive (3). The percentage of positive structures and the average score were computed for pulmonary muscular arteries and bronchial structures in each subject. Immunofluorescence experiments were performed to confirm the exact localization of PARC protein in pulmonary and intercostal arteries with a high PARC expression previously observed by immunohistochemistry experiments.

### Vascular morphometry

The histological and morphometric characteristics of both pulmonary muscular arteries and intercostal arteries were analyzed as described in detailed previously [10], following stereological methods for sampling and fixation for vascular structure evaluation [13]. In brief, tissue was stained with haematoxylin & eosin and orcein to differentiate the internal elastic lamina (IEL) and external elastic lamina (EEL). Vessel cross-sections of intercostals arteries and only pulmonary muscular arteries with an external diameter of 100 to 500 micras were considered in the analysis. Using a computerized image analyzer, the areas occupied by the lumen, the intima and the muscular layer were expressed as a percentage of the total area encompassed by the EEL. The degree of intimal thickening was defined by the percentage of intimal area (%IA = 100X intimal area/ measured total area or area encompassed by the EEL).

### Quantitative real-time-PCR

Quantitative real-time-PCR (qRT-PCR) was performed to determine the gene expression of PARC in lung and IC artery tissue samples. Total RNA was isolated using trizol reagent (Life technologies). Genomic DNA digestion and RNA purification was performed with the DNasa I amplification grade kit (Life technologies). Total purified RNA (1 μg) was reversely transcribed into complementary DNA (cDNA) using the High capacity cDNA kit with RNAse inhibitor (Applied Biosystems, Foster City, CA, USA). Onemicroliter of cDNA was used to perform qRT-PCR using commercial inventoried Taqman assays for PARC (Applied Biosystems Taqman Assay, Hs00268113_m1).qRT-PCR reactions were carried out using the ABI Prism 7900HT Real Time PCR System (Applied Biosystems). Data were collected using SDS software v2.4 (Applied Biosystems) and analyzed by the comparative Ct (ΔΔCt) quantification method using Expression Suite v1.0.3 software (Applied Biosystems). The relative expression of PARC was determined using 18S mRNA (Taqman Assay, Hs03928985_g, Applied Biosystems) as an endogenous control. A common calibrator for each plate was used. Data are reported as a fold change ratio (RQ) of mRNA of PARC from each tissue sample.

### Enzyme-linked immunosorbent assay

To determine circulating levels of PARC in all subjects; an ELISA kit was employed (AB100620-MIP4, Abcam) following the manufacturer’s instructions. Samples were assayed in duplicate. A400 fold serum dilution was used for all samples. The sensitivity of the test was 2pg/ml.

### Study endpoints and sample size calculation

The primary endpoint of this study was the difference in the lung protein content of PARC in patients in the COPD group compared to those in the NOS group. Assuming a standard deviation of 0.22 in band density, a sample size of 16 subjects per group was needed to detect a minimal difference of 0.20 between groups; with 80% power and a two-tailed p-value less than 0.05. Considering an approximate 20% dropout rate (e.g. inadequate samples for measurements), the inclusion of 20 subjects per group was allowed to ensure that data from 16 patients was available for analysis. Secondary endpoints included between-groups comparisons for mRNA expression and immunoreactions for PARC in pulmonary and systemic arteries and their circulating levels of PARC.

### Statistical analysis

For baseline characteristics, continuous variables were expressed as mean ± SD or median and interquartile range whether a normal distribution was assumed or not (Kolmogorov-Smirnov test), respectively. Comparisons of continuous variables were performed with the analysis of variance (ANOVA) method or Kruskal-Wallis test as appropriate, while qualitative variables were compared with the chi-square test or Fisher’s exact test (any expected value <5). An ANOVA method with a general linear model was used to evaluate the primary endpoint and all other between-group comparisons. Adjusted analyses were performed with an ANCOVA method, using unbalanced demographic variables as covariate (gender, pack-years and the presence of diabetes mellitus, P <0.05). Spearman’s correlation coefficients were used to assess the relationship between the percentage of intimal area and PARC immunostaning (labeling intensity score) in pulmonary and IC arteries. A two-tailed P value of <0.05 was considered to indicate a statistically significant difference. Results are reported as least squares mean (LSM) ± standard error of the mean (SEM) for the above detailed analyses. Statistical analysis was performed using PASW Statistics v18.0 software (SPSS Inc., Chicago, IL, USA).

## Results

Consecutive samples from 63 patients undergoing lung resection surgery were included in the study, though six were discarded due to the poor quality or insufficiency of the sample obtained. Therefore, 57 patients were included in the present analysis, 23 COPD subjects, 18 NOS and 16 NS. There were no significant differences in baseline characteristics (Table 1) between groups, except for gender, tobacco exposure and the presence of diabetes; these were therefore included as covariables in all adjusted analyses.

**Table 1:** Baseline characteristics.

Parameters	COPD (N = 23)	NOS (N = 18)	NS (N = 16)	Overall P-value
Male gender, n (%)	21 (91.3)	17 (94.4)	6 (37.5)	<0.001
Age, years	64.4 [60.8–68.8]	61.0 [51.8–68.9]	65.7 [48.0–68.8]	0.75
BMI, kg/m^2^	25.4 [21.8–28.3]	27.1 [24.9–29.9]	26.9 [23.6–30.1]	0.483
Pack-years	42 [35–60]	38 [20–41]	0	<0.001
Systemic hypertension, n (%)	10 (43.5)	8 (44.4)	4 (25)	0.346
Current smokers, n (%)	17 (73.9)	8 (44.4)	0	0.055
Diabetes Mellitus, n (%)	7 (30.4)	8 (44.4)	1 (6.3)	0.044
FEV_1_ Post-BD, % predicted	62.7 [56.1–76.9]	96.7 [85.3–103.6]	103.2 [88.5–118.8]	<0.001
FEV_1_/FVC Post-BD, %	59 [48.4–67]	74.9 [71.4–81.2]	78.2 [74.1–81.9]	<0.001
D_LCO_, % predicted	70.9 [56.8–79.6]	81 [71.5–102.3]	92.2 [76.7–102.3]	0.002
LABA or LAMA, n (%)	13 (56.5)	0 (0)	0 (0)	<0.001
Inhaled CS, n (%)	7 (30.4)	0 (0)	0 (0)	0.003
Leukocytes count, x10E9/L	8.6 [7.6–10]	8.1 [6.9–9.1]	6.4 [5.6–8.3]	0.004
C-reactive protein, mg/L	3.5 [1.4–9.5]	2.4 [1.0–10.1]	1.2 [1.0–2.7]	0.240
Fibrinogen, g/L	3.2 [2.7–3.4]	3.1 [2.6–3.8]	2.8 [2.3–3.7]	0.696

Data are presented as median [25th-75th percentile]. The reported p-value comes from the overall comparison with ANOVA method or Kruskal-Wallis test as appropriate, while qualitative variables were compared with chi-square test or Fisher’s exact test (any expected value <5). COPD: Chronic Obstructive Pulmonary Disease, NOS: non-obstructed smokers, NS: never-smokers, BMI: body mass index, FEV_1_: forced expiratory volume in one second, BD: bronchodilator, FVC: forced vital capacity, DLCO: diffusing capacity of the lungs for carbon monoxide, LABA: long acting β-agonists, CS: corticosteroids.

### Protein expression analysis by western blot

**Fig 1:**
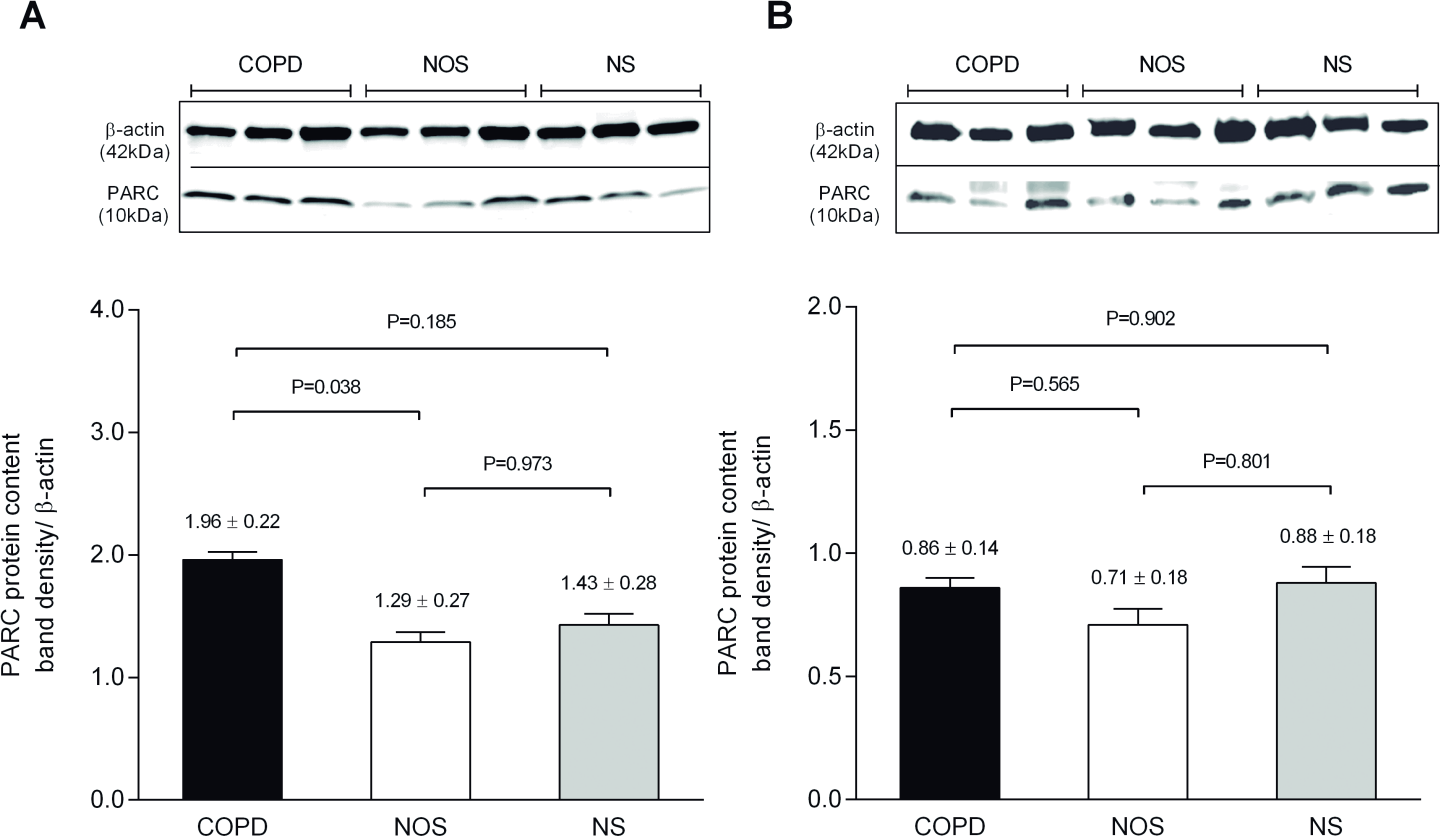
Western blot analysis for PARC. Upper panel: representative membranes of the Western blotting in homogenized lung tissue (A) and intercostal arteries (B). Bands at 37kD and 10kD are consistent with the size of β-actin and PARC respectively. Lower panel: (A) Band density analysis of 33 lung samples (12 COPD, 11 NOS and 10 NS). Of note, protein content was significantly increased in the COPD group compared to NOS. (B) Band density analysis of 28 intercostal artery samples (12 COPD, 8 NOS and 8 NS). There were no differences in PARC content between groups. Data are presented as LSM ± SEM. The reported p-value comes from the pair wise comparison with a general linear model using as covariables: gender, pack-years and the presence of diabetes mellitus. COPD: Chronic Obstructive Pulmonary Disease; NOS: non-obstructed smokers; NS: never-smokers.

Total PARC content in lung and IC artery was measured by western blot. In the lung, protein band density was significantly increased in the COPD group compared to NOS (1.96 ± 0.22 vs. 1.29 ± 0.27, P-adjusted = 0.038). No differences were observed in other between-group comparisons (Fig 1). In the case of IC arteries, PARC content was similar in all groups as shown by the densitometric analysis of the bands (Fig 1).

### Protein expression and cellular localization of PARC

**Fig 2:**
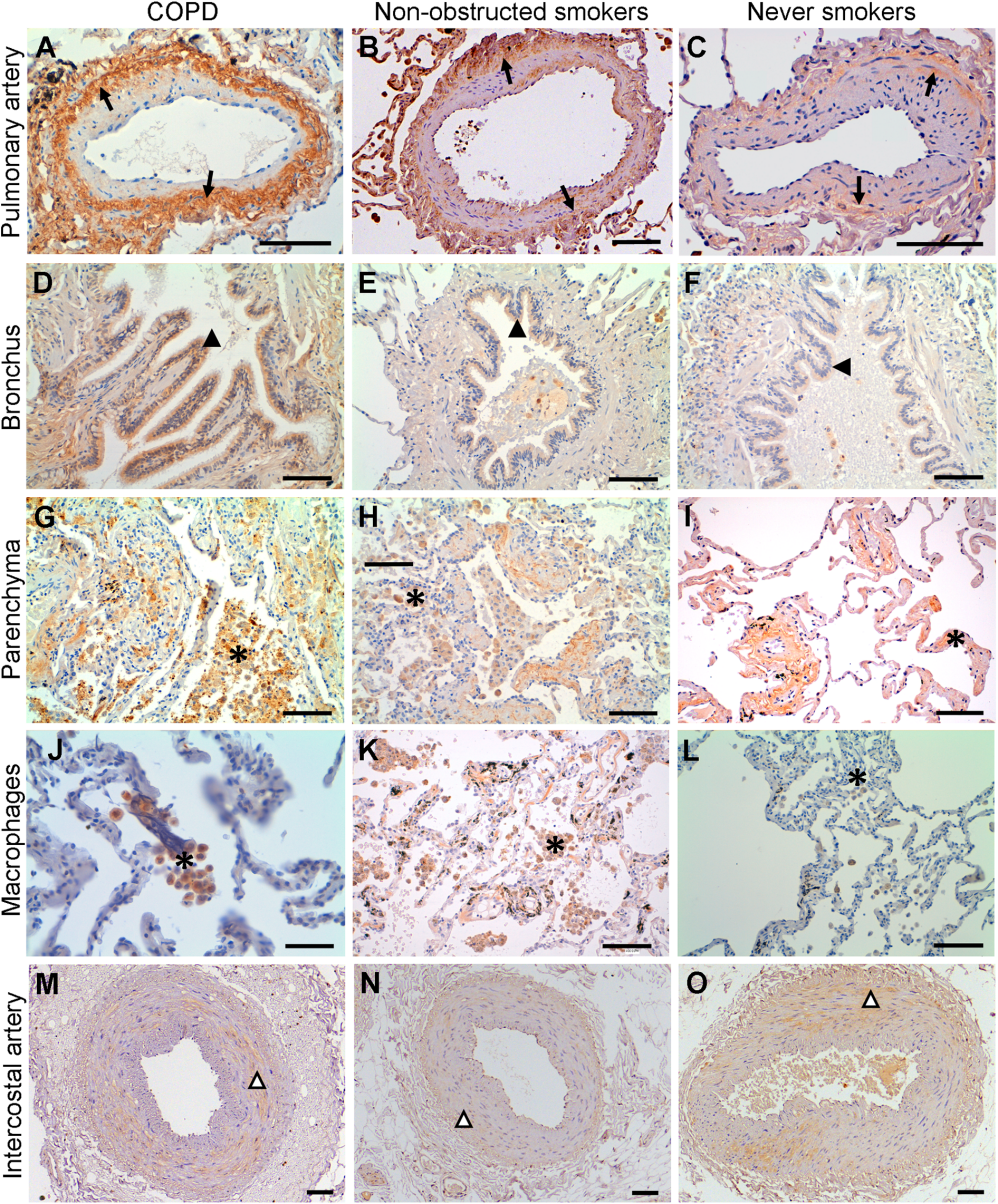
Immunolocalization of MIP-4/PARC. MIP-4/PARC was immunodetected in pulmonary muscular arteries (A-C), bronchus (D-F), alveolar parenchyma (G-I) and macrophages (J-L) as well in the intercostal arteries (M-O) of all biological groups. In pulmonary muscular arteries, MIP-4/PARC was mainly expressed in the muscular layer (arrows). Note that MIP-4/PARC expression is stronger in COPD. In bronchus, MIP-4/PARC was immunolocalized in epithelial cells (filled arrowheads) and its expression was similarly in all biological groups. The immunoreactivity of MIP-4/PARC was higher in the parenchyma of COPD patients. It is show in the magnification images of parenchyma (J-L), labeled macrophage rich areas (asterisks). In the intercostal arteries, MIP-4/PARC expression was mainly localized in the muscular layer (empty arrowheads) but no expression differences between groups were found. Images are representative histological slides from n = 23 COPD, 18 non-obstructed smokers and 16 never smokers. Scale bars = 100 μm except for images in (J-L), where they are 50 μm.

**Fig 3:**
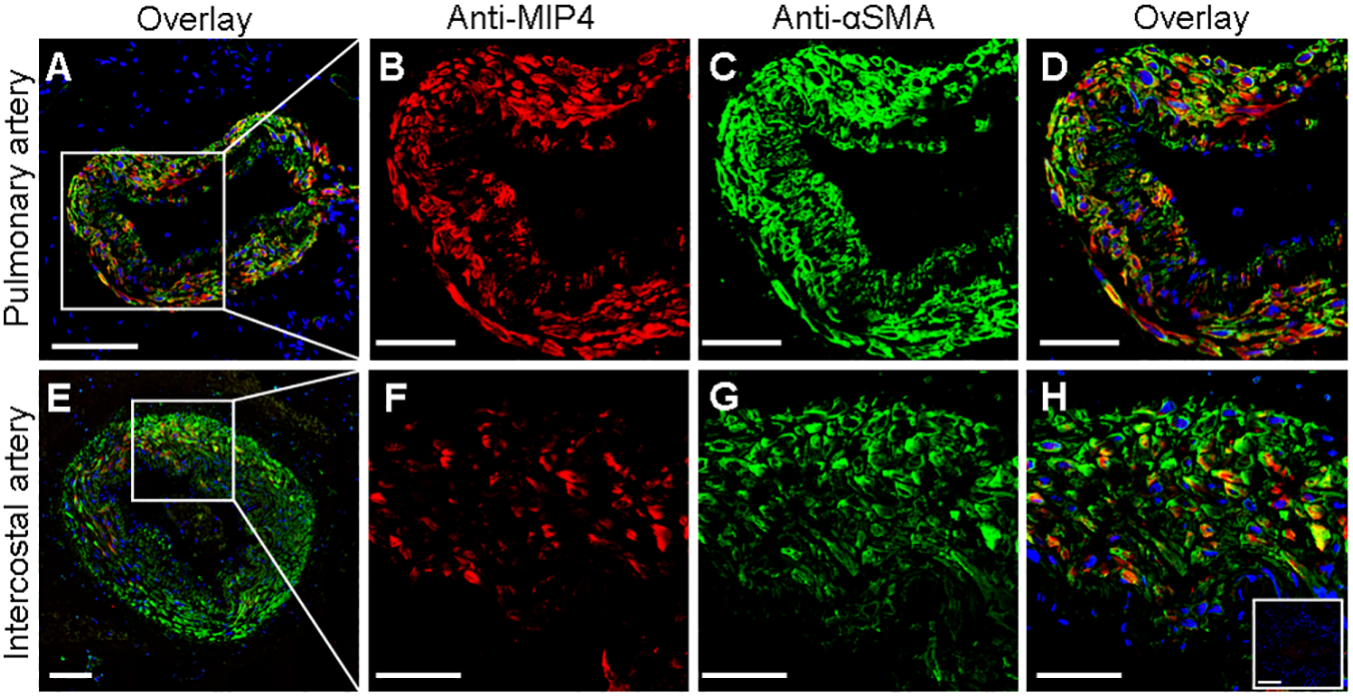
Colocalization of MIP-4/PARC and alpha smooth muscle actin (αSMA) in arterial tissue. Representative confocal fluorescence images of human pulmonary muscular (A) and intercostal (E) arteries labeled with antibodies against MIP-4/PARC (red) and αSMA (green). Nuclei were stained in blue. Higher-magnification (x40 oil lents) of representative images shows that MIP-4/PARC (B and F) and αSMA (C and G) were predominantly localized in the media layer of pulmonary and intercostal arteries. Overlay images show, in yellow, a partially colocalization between MIP-4/PARC and αSMA (D and H). Inset in H corresponds to control experiments performed with secondary antibodies alone. Scale bars = 50 μm except for images A and E, where they are 100 μm.

In order to further characterize the protein expression of PARC in lung and IC artery tissue samples, we carried out immunohistochemistry experiments (the results of which are summarized in Table 2). In the lung, PARC was predominantly immunolocalized in the SMC layer of the pulmonary muscular arteries and in the alveolar parenchyma (mostly in macrophage-rich areas), with a mild expression in bronchial structures (Fig 2). In the comparisons between groups, the percentage of positive pulmonary arteries with strong immunostaining at the muscular layer was higher in the COPD group compared to other groups (Table 2). In IC arteries, PARC was also found to be expressed predominantly in SMC, thoughno labeling differences were observed between groups (Fig 2). Notably, inflammatory cells were not observed in any layer of the IC arteries evaluated by immunohistochemistry. Additionally, the presence of PARC in the SMC layer of both pulmonary and IC arteries was confirmed by double immunostaining performed with anti-MIP-4 and anti-αSMA antibodies. A partial tissue colocalization between PARC and α-SMA is shown in Fig 3.

**Table 2:** Protein expression of PARC in lung tissue and intercostal arteries, according to groups.

Tissue	Parameters	COPD	NOS	NS	Overall P-value
Lung					
	Number of arteries measured in each subject	10.2±1.6	12.4±1.6	9.9±1.9	0.365
	% Positive immunoreaction	72.3 ± 8.1	57.6 ± 8.1	42.9 ± 9	0.063
	Endothelial layer *	0.02 ± 0.03	0.07 ± 0.03	0.08 ± 0.03	0.443
	Intimal thickening *	0.20 ± 0.07	0.13 ± 0.07	0.16 ± 0.08	0.772
	Muscular layer *	1.23 ± 0.15	0.86 ± 0.15	0.61 ± 0.16	0.023
	Adventitial layer*	0.15 ± 0.08	0.1 ± 0.08	0.05 ± 0.09	0.68
	Alveolar parenchyma*	1.33 ± 0.13	1.13 ± 0.13	0.83 ± 0.15	0.059
	Bronchial structures				
	% Positive immunoreaction	79.9 ± 9	59.9 ± 8.7	68.7 ± 9.4	0.293
	Bronchial epithelium*	0.59 ± 0.12	0.41 ± 0.12	0.6 ± 0.12	0.441
	SEBM*	0.1 ± 0.06	0.05 ± 0.06	0.03 ± 0.06	0.7
	Connective tissue*	0.49 ± 0.14	0.53 ± 0.14	0.3 ± 0.15	0.511
	ASM*	0.1 ± 0.08	0.21 ± 0.08	0.03 ± 0.08	0.242
Intercostal artery					
	Endothelial layer*	0.38 ± 0.2	0.31 ± 0.23	0.33 ± 0.24	0.975
	Intimal thickening*	0.31 ± 0.16	0.15 ± 0.18	0.42 ± 0.19	0.597
	Muscular layer*	1.19 ± 0.23	1.08 ± 0.26	1.58 ± 0.27	0.362
	Adventitial layer*	0.56 ± 0.17	0.54 ± 0.19	0.75 ± 0.2	0.694

Data are presented as LSM ± SEM. The reported p-value comes from the overall comparison with ANCOVA method with a general linear model using as covariables: gender, pack-years and the presence of diabetes mellitus.

*Label intensity was scored as negative (0), mild (1), moderate (2), and strongly positive (3). The percentage of positive structures and the average score were computed for pulmonary muscular arteries and bronchial structures in each subject. COPD: Chronic Obstructive Pulmonary Disease; NOS: non-obstructed smokers; NS: never-smokers.

### Relationship between the vascular morphometry and the immunohistochemical expression of PARC

**Fig 4:**
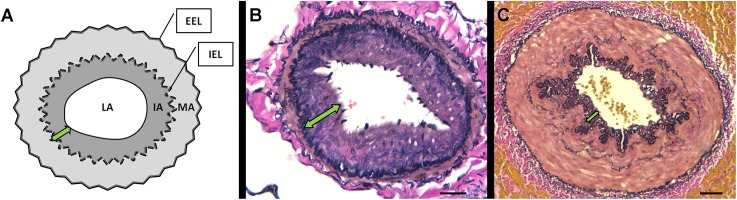
Morphometric and histologic studies. (A) Illustration of the methods used in morphometric analyses. The area enclosed by the continuous black line is the lumen area (LA), the area enclosed by internal elastic lamina (IEL), except for the LA, is the intima area (IA), and the area enclosed by the external elastic lamina (EEL) and IEL is the media area (MA). (B) Elastin-orcein stain of representative muscular pulmonary artery, scale bar = 20μm.(C) Elastin-orcein stain of representative intercostal artery, scale bar = 100 μm. The double-headed green arrows shows intimal thickening (IT) measured by the percentage of intimal area (%IA = 100X intimal area/ measured total area or area encompassed by the EEL). Note the difference between the IT of pulmonary (more remodeled arteries) and intercostal arteries.

According to morphometric analysis, the percentage of the intimal area of pulmonary muscular arteries was significantly increased in the COPD group compared with the NOS and NS groups. However, in the case of the IC arteries, this intimal thickening shows a numerically increasing trend in the COPD group compared to the NOS and NS groups, though it did not attain statistical significance. In both types of arteries, there were no differences in the thickness of the muscular layer between groups. Data relating to morphometric measurements are shown in Table 3. In addition, representative microphotographies of pulmonary and intercostal intimal thickening are shown in Fig 4. In the pulmonary arteries, a significant correlation between the percentage of intimal area and PARC expression in the muscular layer was found (Spearman’s rho = 0.39, p = 0.032) but not in the intimal layer. In the IC arteries no correlations between intimal thickening and the protein expression of PARC were detected (data not shown).

**Table 3:** Morphometric parameters of pulmonary muscular arteries and intercostal arteries.

	COPD (N = 23)	NOS (N = 18)	NS (N = 16)	Overall P-value
Pulmonary muscular arteries				
Total area, mm^-2^x10^-3^	64 [49.8–99.1]	82.7 [64.9–117.4]	75 [67.7–83.4]	0.174
Diameter, μm	294.2 [256.2–362.1]	329.1 [305.7–404.1]	330.6 [317.6–356.1]	0.15
Lumen area %*	25.3 [20.6–34.5]	34.8 [27.4–39.9]	40.2 [29–43]	0.045
Intimal area %*	38.8 [32.2–42.8]	30.5 [21.6–35.1]	22.5 [17.7–37]	0.02
Muscular area %*	33.9 [31.7–38.7]	36.5 [29.6–41.6]	38.2 [34.6–42]	0.519
Intercostal arteries				
Total area, mm^-2^x10^-3^	239.2 [161.7–336.8]	214.6 [145.6–270.2]	200 [142.3–273.5]	0.483
Diameter, μm	556.8 [485.4–668.4]	519.8 [440.1–612.4]	514. [429.6–624.1]	0.515
Lumen area %*	18.7 [15–23.7]	21.3 [12.9–28.3]	26.6 [12.4–34.3]	0.518
Intimal area %*	16.4 [10.6–18.1]	11.9 [11–19.1]	11.8 [8.6–21.2]	0.547
Muscular area %*	65 [59.9–70.7]	64.3 [48.8–68.8]	61 [50–69.3]	0.372

Data are presented as median [25th-75th percentile]. The reported p-value comes from the overall comparison with Kruskal-Wallis test.

*The areas occupied by the lumen, the intima and muscular layer were expressed as a percentage of the total area encompassed by the external elastic lamina. COPD: Chronic Obstructive Pulmonary Disease, NOS: non-obstructed smokers, NS: never-smokers.

### Gene expression in lung tissue and intercostal artery tissue

**Fig 5:**
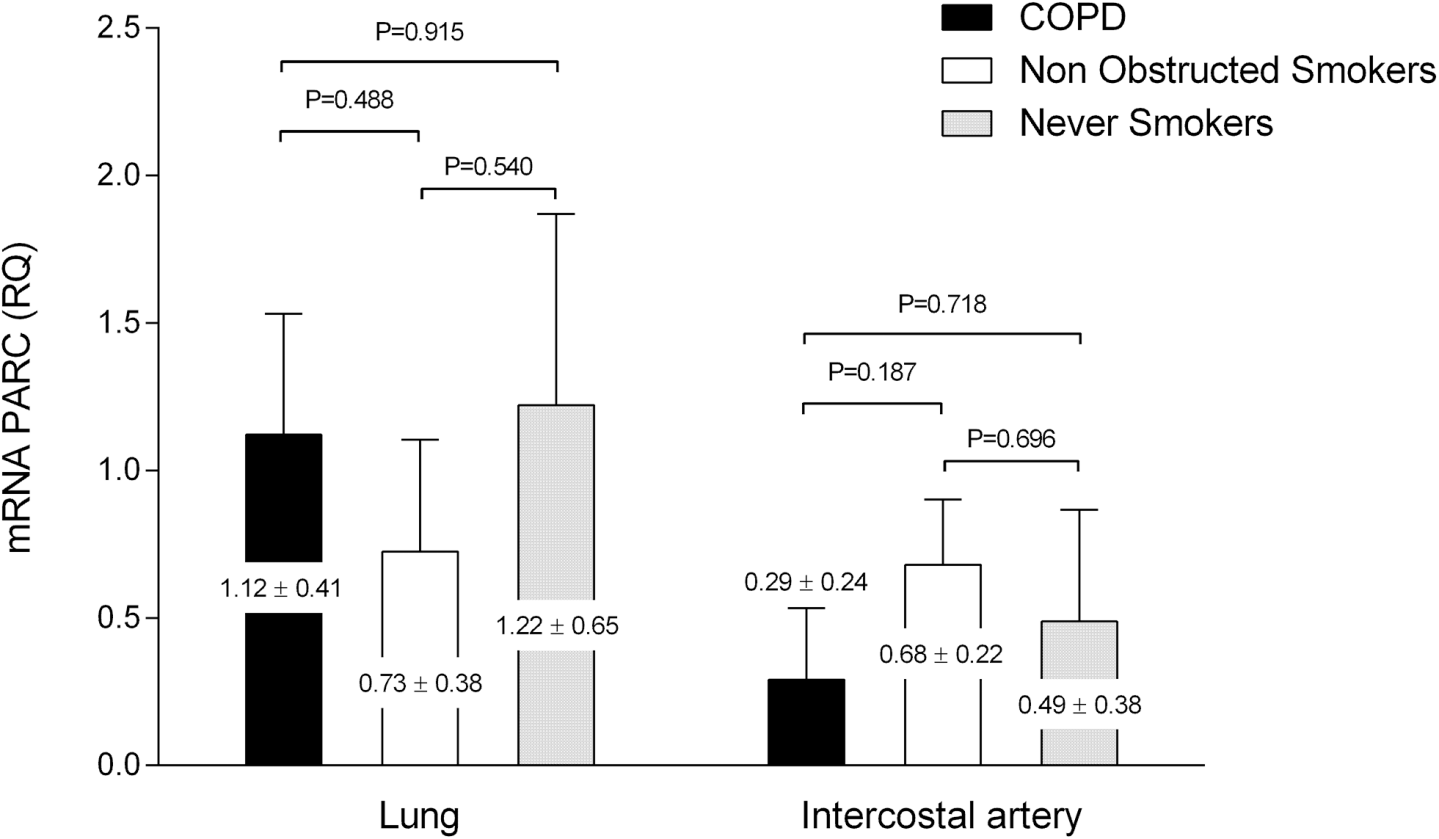
PARC gene expression in according to groups. Data of the PARC mRNA expression (fold change) in lung tissue and intercostal arteries according to groups. No significant differences were found between groups. Data are presented as LSM ± SEM. The reported p-value comes from the pair wise comparison with a general linear model using as covariables: gender, pack-years and the presence of diabetes mellitus.

No differences in the gene expression for PARC measured by the fold change of mRNA were observed between groups in lung tissue or in systemic arterial tissue in the analysis. In the overall population, the mRNA for PARC was constitutively expressed in lung samples (mRNA fold change of 1.02±0.18). However in the intercostal arteries, the expression was low compared to the endogenous control gene 18S (mRNA fold change of 0.48±0.12). Analysis of gene expression by groups is shown in Fig 5.

### Circulating levels of PARC and inflammatory parameters

The circulating level of PARC was also measured by ELISA in the serum of all subjects. PARC concentrations were numerically higher in the COPD group but no statistically significant difference was observed in the comparisons between groups (100.4±19.7 for COPD, 96.6±19.8 for NOS, and 60.8±37.7 pg/ml for NS respectively, *P* overall = 0.718). In the present study, serum levels of C-reactive protein (CRP) and fibrinogen (Table 1) was determined as a measure of systemic inflammation. In order to explore the relationship between systemic inflammation and PARC expression, correlations analyses were performed between these inflammatory parameters and gene, protein and serum expressions of PARC. Of note, a weak correlation between CRP and PARC immuno-staining in alveolar parenchyma and fibrinogen and PARC gene expression at intercostal tissue was found (Spearman’s Rho = 0.36, p = 0,036 and Spearman’s Rho = 0.43, p = 0.022, respectively).

## Discussion

The possible role of PARC in the pathophysiology of COPD has not been fully elucidated. In that sense, the results of the present study contribute to existing knowledge of the protein and gene expression of PARC in lung tissue and in the systemic vascular compartment in patients with COPD. The relevance of inflammation in the context of this chronic disease is beyond doubt [2,11]. This lung inflammation is characterized by both innate immunity (alveolar macrophages, neutrophils, dendritic cells, mast cells, eosinophils, natural killer cells) and adaptive immunity (T- and B-lymphocytes) [14]. However, alveolar macrophages appear to play a key role in orchestrating the inflammatory response [14] by secreting chemokines to attract immune cells, and in the case of PARC, T- lymphocytesin particular, from circulation and to the lung [8,9,15]. Based on that knowledge, we hypothesized that PARC is involved in the pro-inflammatory mechanisms of COPD. We report for the first time an increased protein expression of PARC in the lung tissue of COPD patients compared with non COPD subjects, suggesting a relationship between this chemokine and the development of the disease. These results are consistent with those of other studies of chronic lung diseases such as pulmonary fibrosis in relation to inflammation and disease activity [15–19]. However, the results of the present study do not suggest a higher mRNA PARC expression in both tissues (lung and intercostals arteries) in COPD subjects compared to non-COPD subjects. Also, we did not find any correlationship between gene and protein PARC, suggesting the existence of other possible mechanisms that dissociate the expression of mRNA into protein: spanning the transcription, processing and degradation of mRNAs to the translation, localization, modification and programmed destruction of the proteins themselves [20–21]. Nevertheless, the protein abundances observed reflect a dynamic balance among these processes [21].

Of note, we report through immunohistochemical analyses that this increased lung protein expression may occur especially in the SMCs of pulmonary muscular arteries and that this high expression is correlated with the severity of remodeling (intimal thickening). This suggests that the SMCs in the medial layer could be another source of PARC and that chemokine could be a mediator in these vascular changes. In studies of primary pulmonary hypertension, a profound pulmonary artery remodeling has been described that includes significant fibro-proliferative and inflammatory changes to the entire vascular wall [22]. These findings support the idea that pulmonary hypertension results from a multistep process driven by the reprogramming of the gene-expression patterns that govern changes in cell metabolism, inflammation, and proliferation. Along this line, SMCs phenotypic changes have been described, specifically in the lung vascular remodeling of COPD [23] and in other primary pulmonary vascular diseases [22,24].

Previous studies described an increased expression of PARC in the human atherosclerotic plaques associated with the extent of changes and colocalizing with CD68-macrophages [25–26], with an approximate 100-fold increase in Types II and V lesions compared to normal aortal tissue [25–26]. Therefore, because the COPD population has a clear risk of cardiovascular events and a higher prevalence of subclinical atherosclerosis [27–29], it was thought that one relevant aspect would be the evaluation of PARC expression in the intercostal arteries as a representation of systemic circulation in COPD subjects. However, our results fail to demonstrate differences between groups in PARC expression (both gene and protein) in IC arteries. These findings could be explained by the low inflammation and poor remodeling observed in the IC arteries compared to the pulmonary muscular arteries, where remodeling is more severe. Moreover, in our study the pattern expression of PARC in the IC arteries was predominantly observed in SMCs at the medial layer. These results are in agreement with other studies reporting the presence of chemokines such as MCP-1, MCP-4, and RANTES expressed in the SMCs of atherosclerotic vessels [30–33]. Although the expression of PARC does not appear to be enhanced in the initial vascular remodeling changes of IC arteries in patients with COPD, its possible role in the pathology of advanced vascular disease cannot be discarded, and has been described previously in the context of atherosclerosis [26].

Regarding the use of serum levels of PARC as a biomarker of activity in COPD, there are two large cohorts of COPD subjects (Lung Health Study and ECLIPSE with 4,825 and 1,809 subjects, respectively), which found that PARC was associated with mortality [11–12]. Furthermore, in the context of abdominal aortic aneurysms, PARC (both circulating levels in peripheral blood and gene expression) was associated with aortic lesions with a potential rupture risk [34]. In that sense, PARC could be useful as a serum marker of cardiovascular events in patients with vascular disease [34–35]. These data are in line with the results obtained in the present study, in which circulating levels of PARC have a numerical tendency to be higher in COPD subjects compared to other groups, though this trend did not reach statistical significance due to the high biological variability between subjects and the limited number of cases available for analysis.

Several limitations of this study need to be discussed. Firstly, the poor representation of female gender in the COPD and NOS groups due to the baseline characteristics of our population (patients with lung carcinoma and a major smoking habit are mostly male patients). This gender misbalance makes it difficult to draw conclusions about gender beyond spurious associations. Secondly, the population of the study has primary, treatable lung cancer; therefore lung cancer could be a possible introduced bias. However, we are assuming that any bias introduced because of lung carcinoma would be the same across all the subjects, since all the subjects included in the study suffer from lung carcinoma. Taking into account this limitation, we are able to compare PARC expression in order to find differences between COPD subjects and non-obstructed smokers, assuming that PARC expression in each group could be similarly influenced by the presence of lung cancer. It is important to consider that it would be impossible to obtain the demographic data, pulmonary function test and all the tissue specimens required in this study from subjects if they were not indicated for surgery. Tercially, the negative results found in circulating levels of PARC expression should be taken with caution, since a possible underpowered analysis due to small sample size and important biological variability could limit our results. Finally, due to the study’s observational design, causal or strong conclusions cannot be drawn beyond observing an association between the presence of COPD and the expression of PARC.

## Conclusions

In conclusion, the results of the present study suggest that PARC could have a relevant role in the development of vascular abnormalities in COPD, specifically in the lung, where the remodeled pulmonary vessels are present. However, other studies, especially experimental approaches, are needed to confirm these findings.
